# Relative Importance of Modularity and Other Morphological Attributes on Different Types of Lithic Point Weapons: Assessing Functional Variations

**DOI:** 10.1371/journal.pone.0048009

**Published:** 2012-10-19

**Authors:** Rolando González-José, Judith Charlin

**Affiliations:** 1 Unidad de Investigación en Diversidad, Sistemática y Evolución. Centro Nacional Patagónico-CONICET, Puerto Madryn. Argentina; 2 Instituto Multidisciplinario de Historia y Ciencias Humanas-CONICET, Universidad de Buenos Aires, Ciudad Autónoma de Buenos Aires, Argentina; New York State Museum, United States of America

## Abstract

The specific using of different prehistoric weapons is mainly determined by its physical properties, which provide a relative advantage or disadvantage to perform a given, particular function. Since these physical properties are integrated to accomplish that function, examining design variables and their pattern of integration or modularity is of interest to estimate the past function of a point. Here we analyze a composite sample of lithic points from southern Patagonia likely formed by arrows, thrown spears and hand-held points to test if they can be viewed as a two-module system formed by the blade and the stem, and to evaluate the degree in which shape, size, asymmetry, blade: stem length ratio, and tip angle explain the observed variance and differentiation among points supposedly aimed to accomplish different functions. To do so we performed a geometric morphometric analysis on 118 lithic points, departing from 24 two-dimensional landmark and semi landmarks placed on the point's contour. Klingenberg's covariational modularity tests were used to evaluate different modularity hypotheses, and a composite PCA including shape, size, asymmetry, blade: stem length ratio, and tip angle was used to estimate the importance of each attribute to explaining variation patterns. Results show that the blade and the stem can be seen as “near decomposable units” in the points integrating the studied sample. However, this modular pattern changes after removing the effects of reduction. Indeed, a resharpened point tends to show a tip/rest of the point modular pattern. The composite PCA analyses evidenced three different patterns of morphometric attributes compatible with arrows, thrown spears, and hand-held tools. Interestingly, when analyzed independently, these groups show differences in their modular organization. Our results indicate that stone tools can be approached as flexible designs, characterized by a composite set of interacting morphometric attributes, and evolving on a modular way.

## Introduction

The study of prehistoric weapons, especially how they were used, is a complex task for archaeologists because the overall technical system, which is mostly composed of perishable materials, is rarely preserved in the archaeological record. The weapon points, mainly the lithic ones, are typically the only elements recovered, and a weapon system functional is usually estimated by analyzing their morphometric attributes.

Given that specific combinations of physical properties on a point can be seen as an “optimum” that provide a relative advantage to perform a given function, examining design variables and raw materials properties from a mechanical physic and optimal engineering point of view is a useful approach to estimate the past function of weapon points [1]–[8]. For example, an object intended to fly long distances from the propulsion system to the prey must move across some design thresholds in order to guarantee minimum values of aerodynamics, penetration, weight, surface of contact with the prey, hafting attachment, etc. More precisely, when a projectile is thrown, the air resists its motion and this resistance increases with velocity [2]. In vacuum conditions, a projectile will attain the maximum range if it is launched at an angle of 45° to the horizontal [2], but in fact the range of, for example, an arrow in the air varies from 60 to 90% of its theoretical range in a vacuum (Pratt 1976 in ref. 2). In aerodynamics, the resistance of the air on a projectile is called drag, and it depends on size and shape of the projectile and on the dynamic pressure (a relationship between the air density and the throw velocity) [2], [8]. In other words, the resistance that a fluid opposes to the movement of the projectile is proportional to its surface, shape, velocity and fluid density [8]. As a practical conclusion, the air resistance makes the projectile to lose energy, and hence power of impact, as a function of distance [8].

Conversely, a point aimed to function as a hand-held tool (e.g. a thrusting spear) may require different attributes in order to achieve a functional optimum (or at least minimum) performance. For instance, because a thrusting spear is not a flight weapon, only penetration and durability are operative in its design [3].

The archaeological record provides a complicated picture of tool use, where some points can be viewed as functional optima in mechanical terms, some of them as suboptimal, and many of them present overlapping attributes that complicate its classification under functional criteria. Furthermore, reutilization and recycling of damaged larger points in order to be used as smaller, new ones or else as other kinds of tool derive on a palimpsest of traits that further obscures the using of morphological attributes to classify points into solid, discrete functional categories. In this regard, we have recently suggested a major incidence of reduction practices on shape rather than on size, and in the blade rather in the stem of points [9]–[10]. However, and besides the effects of reduction, different southern Patagonian Late Holocene lithic point types can be distinguished in terms of size and stem shape. Thus, even though successive cycles of use, damage and resharpening have a great influence on point's size and shape, resharpening techniques are specific enough to maintain size and shape differences between types, an issue that is probably related to functional requirements [9]. Since the nature of tool making, using, and reusing affects at the same time the different design parameters that determine the tool optimal function, the investigation on how the different physical attributes contribute to discrimination among arrows, thrown spears, and hand-held weapons is of utility to understand the relative importance of the different traits in the context of functional demands.

Regarding the behavior of blade and stem, an interesting question arises around the modular nature of both structures, commonly assumed as techno-functional units. Modularity is based on the idea of a map of connections or interactions among the components of a system in which some areas have denser internal versus external connections [11]–[14]. Artifact systems tend to be organized into clusters, or modules, which consist of parts that are integrated tightly by many or strong interactions and which are relatively independent from other modules because there are fewer or weaker interactions between them. But besides these covariational properties, from a process-oriented perspective, modules are characterized as units of interacting components that operate in an integrated (interdependent) but relatively context-insensitive manner, and therefore behave relatively invariantly in different contexts [15], [16]. Thus, modules can be defined focusing on their internal relations (e.g. shape and size attributes of the blade or the stem), but also considering their autonomy, concerning their external relations (e.g. the blade in relation to the rest of the point) [16]. An important derivation of modularity on stone tools is that modularization is economically advantageous as it facilitates designing, constructing, and maintaining artifacts [1], [17], [18]. Also, it is preferable on certain behavioral and ecological context as those of unpredictable but continuous resource availability and low failure costs [1].

Modular systems can evolve from different starting points by changes going in opposite directions: by parcellation of a highly integrated system, or by integration of existing systems [19]. The mechanisms producing modularity are usually described as a specialization of existing structures in the case of parcellation, and as assembled processes in the case of integration [20], [18]. Excepting the case of some composite Mode 5 microlithic tools, most of the bifacial stone points can be seen as examples of specialization of pre existing structures (e.g. the initial node). At the level of tool design, different parts of an artifact such as the blade and the stem could be designed as working independently of one another. The “modules” could then be connected and (in theory at least) would function seamlessly, as long as they conformed to a predetermined set of design rules [21].

In consequence, the question arises whether the modular nature of artifacts evolved early on human societies or it's just a characteristic of modern, developed technologies. In pre-industrial technologies such as lithic points, functional demands can differ greatly. For instance, the blade is expected to fulfill the requirements of a projectile on a flying object. On the contrary, the stem attributes are intended to guarantee a proper, solid attachment to the haft. If these divergent functional demands are greater than design constraints, thus depicting a weapon point as a combination of two “nearly decomposable systems”, then one should expect a significant modular behavior of the blade and the stem.

Considering all the above, and using a composite sample of lithic points from southern Patagonia (Argentina and Chile) formed by arrows, thrown and hand-held points classified as such in previous analyses [7], [22], here we focus on three different objectives. First we aim to test if these points can be viewed as a two-module system formed by the blade and the stem. We test this hypothesis considering different types of points and different shape attributes. Secondly, we evaluate which morphological attributes (whole shape, blade shape, stem shape, size, asymmetry, blade: stem length ratio, tip angle) explain greater variance values on the composite sample. Finally, we use the most explanatory variables to verify if the classical typological classification proposed by Bird (refs. 23–25) results on clear-cut separate groups of points.

## Materials and Methods

The sample used here is an extension of the one used on a previous paper, and consists of Late Holocene lithic points (n = 118) from southern Patagonia(southern Santa Cruz Province, Argentina and Magallanes, Chile) [9].

Projectile points were classified as Bird IV–Vtypes by their own discoverers (see ref. 9), except 14 pieces corresponding to sitesstudied by one of us (JC, Cóndor cave 1, Norte 2, Laguna Azul, and Laguna Cóndor) or belonging to previous collections that were reanalyzed to recover geometric-morphometric data. Further details regarding the archaeological, geological, and bio-anthropological context for this sample are provided in ref. 9.

Photographs were taken on completed points by one of us (JC) in surface and subsurface archaeological surveys along the region, and on digitized images of points published in the literature. Lithic points were assigned to two different typological categories according to Bird's [23]–[25] pioneering classification (see a detailed review of this classification system in ref. 9). Classification under the different criteria as well as further qualitative and quantitative data of each point is presented on Table S1.

**Figure 1:**
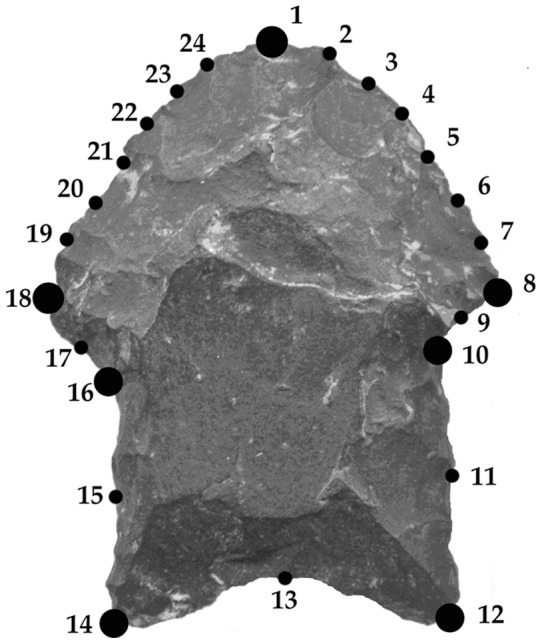
Landmark configuration. Landmarks (large dots) and semi-landmarks (small dots) used in this study.

Only complete, non fragmentary points were taken into account. Small damages (≤3 mm) were tolerated and the shape was rebuilt from the adjacent planes of the piece. Illustrations and photographs were scanned in a digitizing tablet, keeping constant the digitizing scale (100% in cm), resolution (100 dpi), and point orientation (the tip towards the upper border). The raw images were compiled and scaled in the TpsUtil and TpsDig2 programs respectively [26], [27]. A total of seven landmark and 17 semi-landmark coordinates were digitized on the contour of the points in order to achieve a good representation of its size and shape (Figure 1). Landmark configurations were superimposed using a Generalized Procrustes Analysis (GPA, 28, 29), and sliding semi-landmarks were relaxed following the minimum bending energy criterion implemented in the TPSRelw (version 1.45) software [30]. GPA removes the effects of translation, rotation, and scaling [28], [29], [31]. After superimposition, pure shape information is preserved in the specimens' aligned landmarks. Size is calculated as the centroid size, the square root of the summed distances between each landmark coordinate and the centroid [31]. Subsequent analyses were done with the complete, 24-landmark configuration, or else with partial configurations consisting of blade-alone landmarks (1 to 9 and 17 to 24 in Fig. 1), or stem-alone landmarks (10 to 16 in Fig. 1).

The Procrustes superimposed coordinates were further used to obtain angle measurements, proportions, and asymmetry values for each point. Specifically, we computed the tip angle (TA, in plain view), the ratio of blade length to stem length (IBS), and asymmetry values (AS) as indicated in [32]. Tip angle and the index blade-stem length were chosen because previous experimental and comparative studies (reviewed in ref. 9) have shown that, given that the major size and shape changes associated to successive cycles of use and resharpening occur in the point blade, mainly in its length and tip angle [33]–[43], then they can be considered as reliable reduction estimators. In some specific analyses, we attempt to minimize the effects of reduction on shape by regressing the shape coordinates on IBS and/or TA. Departing from previous comparative and experimental studies (see a review on ref. 9), and assuming that IBS or TA are good proxies to represent reduction phases, then the residuals of the regression of shape on IBS/TA can be seen as the portion of shape variation that is preserved in the data when the effects of reduction are removed.

Asymmetry individual scores quantify the individual asymmetries of shape (as deviations from the mean asymmetry) by using Mahalanobis distances, which are scaled relative to the variation of asymmetry in the sample.

The analysis of the point modularity was done considering the hypothesis that the blade and the stem are distinct modules. If this hypothesis is true, each of these regions should be highly integrated internally and relatively independent to the other region. Modularity can be assessed by analyzing covariation among subsets of landmarks [44], [45]. Since a strong covariation within modules does not contribute to covariation between subsets, a weak covariation among hypothetical modules is expected if the subconfigurations of landmarks closely resemble the true ones [44], [45]. Conversely, if the blade and stem do not fit to the true modules, the weak within-module integration contributes to the covariation among sub-configurations that will therefore be stronger. Overall, it is expected that covariation among subsets is weaker for subsets corresponding to the true modules than for other partitions of the landmarks into subsets [45]. To assess the hypothesis of blade versus stem modularity, we computed the multisetRV coefficient [45]. The RV coefficient is a measure of the strength of internal (within module) covariance relative to external covariance. Then, the analysis compares the RV coefficient or multi-set RV coefficient for the partition of the landmark configuration into the hypothesized modules with alternative partitions into subsets of the same numbers of landmarks [45]. The multiset RV coefficient was computed from the Procrustes-aligned coordinates of the landmarks of the blade and stem, before and after correcting for the effects of size, IBS and TA, and for 10,000 random partitions of the total set into random subsets containing the corresponding numbers of landmarks. Size-, IBS- and TA-corrected shape data was obtained by using the residuals of the multivariate regression of the Procrustes aligned shape coordinates against the centroid size, IBS and TA respectively.

The complete configuration, as well as the blade and stem sub-configurations, was submitted to three independent Principal Component analyses (PCA) of shape in order to obtain axes of maximum shape variation for the whole points, the blades, and the stems. The first PCs of each analysis, depicting the main trend of shape variation on each configuration, along with the tip angle (TA), the index blade length/stem length (IBS), the point size (cs), and the asymmetry scores (AS) were collectively submitted to a further Principal Component analysis in order to synthesize data and explore the relative contribution of each trait to the total variation observed in the sample. Since the different attributes are measured on different scales, the matrix of correlation was used as the basis to perform the composite PCA. Even though TA and IBS are indeed shape indicators, we have decided to include it on the composite PCA along with the pure shape variables (first PC of the Procrustes coordinates) because TA and IBS refer to specific shape attributes that have been previously used to control the effects of reduction. There are other portions of shape variation that, with some probability, does not respond to resharpening effects. In consequence, the necessity of maintaining it separated from the complete approach to shape (PCs of shape coordinates) respond to enable comparisons with previous using of TA and IBS as reduction-dependant variables. Note that the intention of the composite PCA is to collectively and simultaneously explore the full shape variation, specific shape variation previously associated to reduction (IBS, TA), asymmetry, and size. Of course, any correlation among the behaviour of these variables will be accounted for the composite PCA, for instance, by sorting correlated traits along the same PC.

## Results

**Figure 2:**
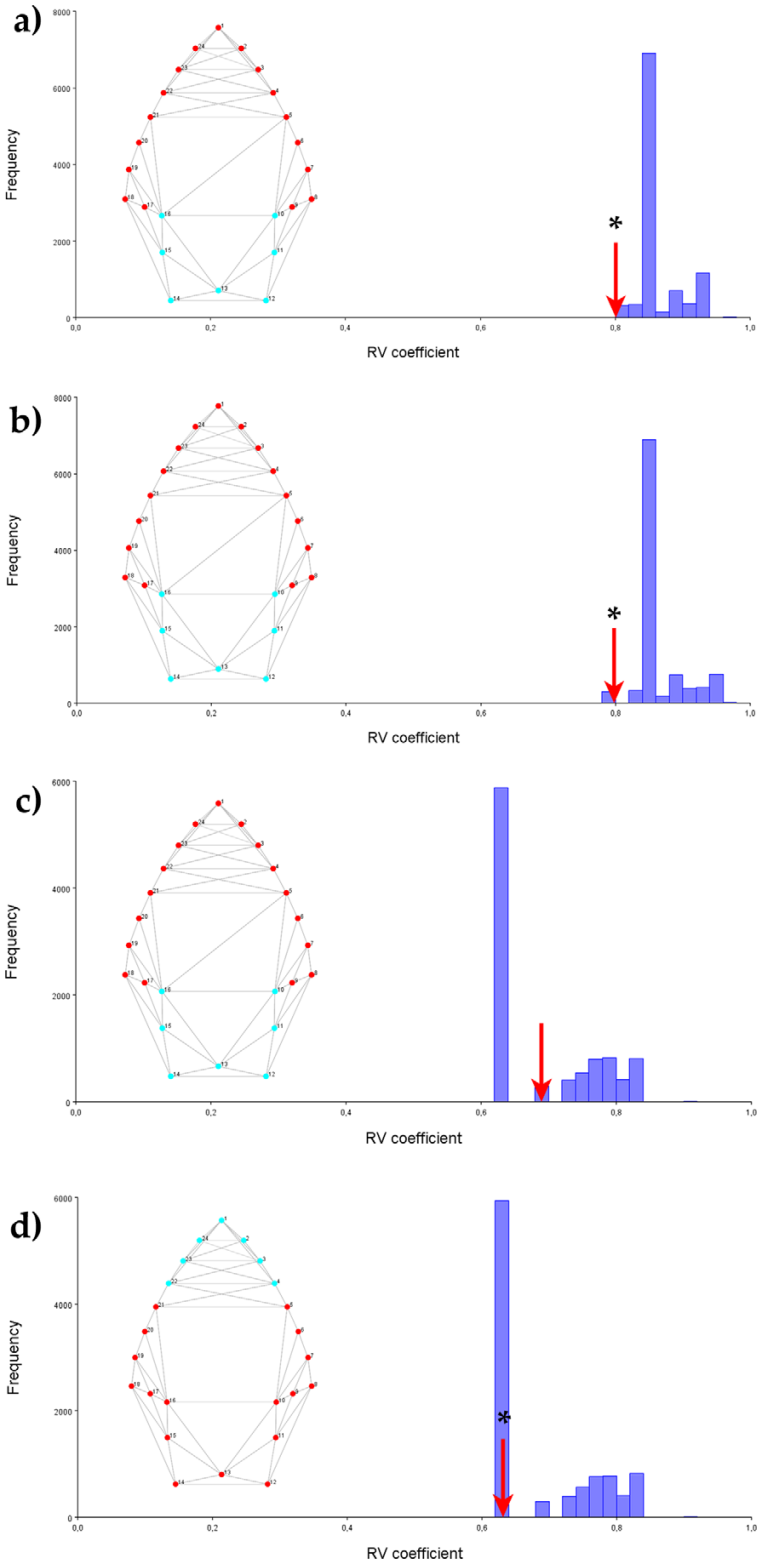
Modularity tests. Tests of modularity using the method of Klingenberg [45] based on 2D landmark data for 118 points. A) Pure shape dataset, blade/stem hypothesis; b) size-corrected dataset, blade/stem hypothesis; c) IBS-corrected dataset, blade/stem hypothesis; d) IBS-corrected dataset, tip/rest of the point hypothesis; The hypothetical modules tested are shown as red versus light-blue landmark configurations. The histograms show the distribution of the RV coefficients obtained from 10000 permutations of possible combinations of contiguous landmarks. The red arrow indicates the observed RV value. Significant modularity hypothesis are marked with an asterisk.

The hypothesis of blade and stem modularity was evaluated by comparing the multiset RV coefficient for this partition of landmarks with alternative partitions into spatially contiguous subsets [45]. For the Procrustes aligned dataset, the multiset RV coefficient is 0.80 (Fig. 2a), and none of the 10,000 random partitions yielded weaker associations among subsets. Because the association between the two subsets is in the lower extreme of the distribution of multiset RV coefficients the hypothesis of modularity is not rejected for this data set.

When the effects of size are removed from the raw data set (by multivariate regression of shape on size), the resulting size-free data set provides a multiset RV coefficient of 0.79 (Fig. 2b) and, again, there were no partitions with RV less than the a-priori hypothesis. Collectively, these results indicate that, in this sample, the blade and the stem can be seen as “near decomposable units”. In addition, it can be said that this modular pattern is preserved after the effects of size are removed from the shape information.

The regression of shape on IBS was significant (p<.0001) and around a 49% of shape variation is explained by IBS. Thus, use and resharpening effects were removed by regressing shape coordinates on the IBS index and using the residual of the regression as IBS-corrected data. The IBS-corrected data was then submitted to the modularity analysis, which yielded a non-significant multiset RV coefficient of 0.68 (Fig. 2c). Thus, the previously reported modularity of the blade and the stem vanishes when reduction effects are removed from the shape data. An alternative partition of landmarks considering a “tip” module (landmarks 1–4 and 22–24) and a “rest of the point” module (landmarks 5–21) resulted on a significant (p<0.00001) multiset RV coefficient of 0.63 (Fig. 2d). The same method applied to Tip Angle-corrected data yielded similar results (not shown). In sum, the correction for reduction effects produced a key change in the covariation pattern of landmarks between the blade and the stem of the point. In other words, when the effects of resharpening are preserved in the shape information, a blade/stem modular pattern is evident. Conversely, when the effects of resharpening are removed, the remaining shape variation is structured into a tip/rest of the point modular pattern.

**Figure 3:**
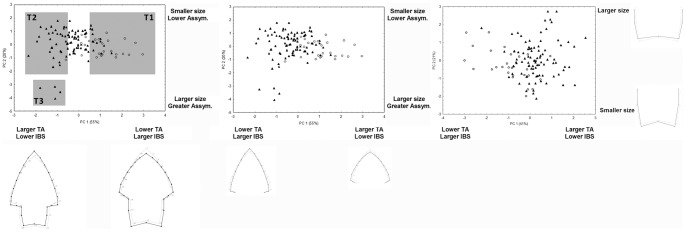
Composite principal component analysis. Scatterplot of the two first principal components performed on the first PC of shape, size, IBS, TA, and asymmetry values. A) Whole point shape analyzed; b) only blade shape analyzed; c) only stem shape analyzed. The color of the points indicate classification of points as Bird IV (solid triangles), Bird V (empty circles) types according to Bird [23]–[25]. T1, T2 and T3 represent arbitrary groupings aimed to repeat the modularity analysis on arrays of points sharing similar attributes.

The PCA computed independently on the whole point, the blade and the stem yielded a first PC explaining 67.1%, 83.7%, and 60.2% of the total variation, respectively. These first PCs of shape (considering the whole point, the blade or the stem), size, IBS, TA, and AS were submitted to a correlation-matrix based Principal Component Analyses in order to detect possible associations among these variables, and also to estimate if putative “natural” grouping of points fit well to the Bird's classification. The results are presented on Table 1 and Figure 3a–c. When the shape of the whole point, along with its size, blade/stem length ratio, tip angle, and asymmetry value is analyzed on the composite PCA (Figure 3a), the first PC is mainly dominated by variations on the shape, TA and IBS (Table 1), and explains a 55.4% of the total variation. Specifically, positive values are represented by elongated blades with narrow stems, small tip angles and large IBS. When the pioneering classification proposed by Bird is observed, it indicates that Bird V or “Ona” points, assumed to be arrows because of their small size, are placed on the right region of the plot. Conversely, Bird IV or “Patagónicas” points, showing shorter blades and wider stems, greater tip angles and lower IBS occupy the negative values across the first PC. The second PC is mainly dominated by size (small points in the positive values), and asymmetry (asymmetric points towards the negative values), and explains a 20.1% of the total variation. Interestingly, all the Bird V and most of the Bird IV points are placed on the positive (small, non asymmetric) end of the PC2, whereas a small sub sample of four Bird IV points (enclosed arbitrarily into an ellipse in Fig. 3a) occupy the negative values of this second PC.

**Table 1:** Results concerning the composite PCA made after size, blade-stem length index (IBS), tip-angle, asymmetry, and shape of the whole projectile (left columns), blade (central columns), and stem (right columns).

	Wholepoint		Blade		Stem	
	PC 1 (55%)	PC 2 (20%)	PC 1 (55%)	PC 2 (21%)	PC 1 (41%)	PC 2 (21%)
cs	−0.288	**−0.671**	0.286	**−0.657**	0.347	**0.610**
IBS	**0.903**	−0.114	**−0.876**	−0.078	**−0.919**	0.018
TA	**−0.928**	0.141	**0.953**	0.139	**0.795**	0.356
As	−0.268	**−0.714**	0.247	**−0.735**	0.382	−0.291
Shape (PC1)	**−0.969**	0.155	**−0.962**	−0.175	0.551	**−0.666**

Correlation values among the first two PCs and the variables are shown. Bolded values represent strong correlations among a variable and a PC. Explained variance of each PC among parentheses.

A similar analysis performed using the blade shape instead of the whole point shape provided very similar results (Figure 3b, Table 1), with shape, tip angle, and IBS dominating the variation across the first PC (54.8% of explained variation) and size and asymmetry contributing mainly to the second axis (20.4% of explained variation).

Finally, the analysis of stem shape plus size, TA, IBS and AS (Figure 3c, Table 1) indicates that stem shape is not as important as blade shape to determine axis of principal variation. In particular, the first PC is dominated by variations on TA and IBS (40.9% of explained variation), and shape is sorted across the second PC (20.5% of explained variation), along with size. The separation between Bird IV and V point types is not as evident as in the previous analyses. Moreover, the cluster of four points observed on the previous two analyses vanishes when the stem shape is considered (Figure 3c).

In sum, three patterns are observable in the composite analyses considering whole (or blade) shape, size, tip angle, blade-stem length ratio and asymmetry. Firstly, it is observable a group of extreme negative values presenting elongated blades with narrow stems, small tip angles and large IBS, clustered into a Type 1 label on Figure 3a. Secondly, there is a group of short blades and wide stems, greater tip angles and lower IBS occupying the negative values of the first PC, and labeled as Type 2 on Figure 3a. Both Type 1 and 2 groups present rather symmetric shapes. Finally, there is a small group of large and asymmetric points on the negative values of the second PC, labeled as Type 3 points. Of course, many specimens cannot be reliably placed into any of these three groups, since they present intermediate attributes probably related to recycling activities and changes in the original functions for which the tools were designed.

**Figure 4:**
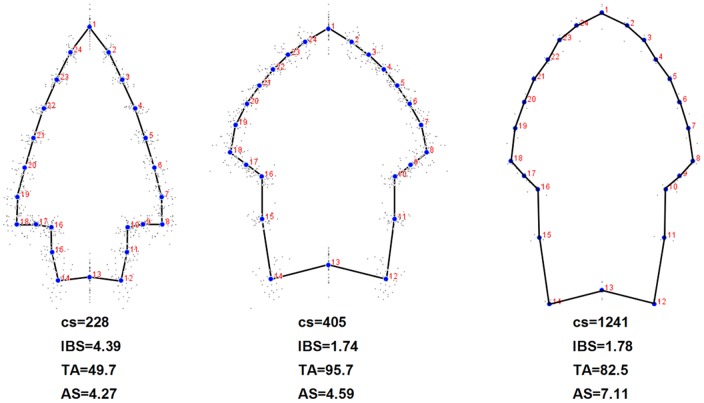
Consensus configurations. Consensus for the Type 1 (left), 2 (center), and 3 (right) groupings derived from the composite PC analyses presented on Fig. 3. Mean values of size (cs), blade-stem length ratio (IBS), tip-angle (TA), and asymmetry (AS) are presented.

The question arises if the modular behavior observed on the whole sample, that is, a blade/stem modular structure that turns into a tip/rest of the point modular pattern when the effects of resharpening are removed, is maintained on the three types observed in the composite PC analyses (Figure 3a). Consensus configurations of the three constructed types are presented on figure 4, and results concerning the modularity analyses are presented in Table 2. Reinforcing the results presented on Figure 3, Figure 4 indicates that there are important differences of shape among the three arbitrary types, but also that the Type 3 points differ much in terms of size and asymmetry. The modularity tests reveal two different patterns. On one hand, Types 1 and 2 depict points with a strong modular behavior of the tip versus the rest of the piece, even when the effects of reduction are eliminated. On the other hand, Type 3 behaves as a blade/stem two-module system, no matter if reduction is controlled or not.

**Table 2:** Results of the modularity analyses made on the three subsamples defined after Figure 3a.

	Blade/stem hypothesis		Tip/rest of the point hypothesis	
Sample	Multiset RV	p-value	Multiset RV	p-value
T1	0.76 (0.77)	0.594 (0.607)	**0.66 (0.76)**	**<0.00001 (0.035)**
T2	0.81 (0.78)	0.643 (0.618)	**0.75 (0.66)**	**0.009 (0.014)**
T3	**0.61 (0.53)**	**<0.00001 (<0.00001)**	0.94 (0.86)	0.416 (0.273)

For each grouping we provide the multiset RV coefficient and associated P-value for two modularity hypotheses: blade/stem and tip/rest of the point. Values in parentheses correspond to results obtained after adjusting for reduction (IBS) effects. Significant RVs are bolded.

## Discussion

It has been argued that variability in stone tools, rather than reflecting the social and cultural groupings of the populations who made them, reflects the demands of the environment and the responses of the populations to those demands within the constraints of raw-material availability, mobility patterns and hunting strategies, among others [46]. Even when stone stools can be seen as simple artifacts, lithic technology was also a complex system of central importance to cultural adaptation during long periods of human evolution. In this sense, stone tools are as complex as most modern technical artifacts. Central to this concept, is the way in which stone tools are designed and maintained throughout their life-cycle, and the potential utility of a modular approach to fulfill the environmental demands, especially in maintainable designs [1]. Modularity means that the parts of the system are grouped in such a way that strong interactions occur within each group or module, but parts belonging to different modules interact only weakly [15], [16], [20]. Applied to a typical stemmed lithic point, it can be translated to the fact that a point is formed by (in principle) two sub units that are not maximally integrated among them (e.g. the blade and the stem). In this regard, lithic points can be intended as modular systems that can evolve by parcellation of a highly integrated system (e.g. the core). Since the maximum possible degree of complexity depends on the number of components of a system and on the number of interactions between them, a limitation of interactions in a system that consists of partly independent subsystems reduces complexity. Exactly such limited interaction among subsets occurs in systems organized in a modular way.

The way in which stone tools are made, through a process of core and flake reduction and thinning, and aimed to work as arrows, thrown spears or hand-held tools is important to determine the modular organization of the tool in its initial design. However, it has been argued that much of the differences among typological elements are the product of different degrees of reduction, and that, for example, a few more blows and one type is transformed into another [9]. A central implication of reduction is that it can alter the initial modular organization of the tools as it is damaged and recomposed and transformed throughout its life-cycle. Our results presented in figure 2 lend support to this notion: on the whole sample, where there is likely an assemblage composed by arrows, thrown spears and some hand-held tools, the general shape of the point can be decomposed onto a blade and a stem two-module system. When the reduction effects are removed, the residual portion of the shape not explained by this practice is, however, arranged into a tip versus rest of the point modular pattern. This result is quite logic, if we consider that the blade is more affected by the reduction and resharpening process, and it would reinforce the previous idea that Late Holocene southern Patagonia point rejuvenation is made without removing the point-haft attachment [47].

The problem arises that on point assemblages as the one studied here, it is likely that different kind of technical weapon systems are present. Since different functions are related to optimal physical properties, the exploration of these traits on a model-bound approach can be of utility to apportion the sample into sub-groupings sharing expected attributes of each specific function (e.g. arrow, thrown spears, and hand-held tools). For instance, a long and narrow blade, a narrow stem, a moderate to low size, a small tip angle, and a symmetric form are preferred traits when building an arrow, since it guarantees low resistance to the air [2], [3], a small and thin attachment region [48]–[50], maximum penetration [3], [51], and proper aerodynamic properties [2], [8].

Since they are also objects aimed to impact and penetrate the preys' body, thrown spears require quite similar attributes, but some authors argue that bilateral symmetry is not as important as in arrows, that the stem is expected to be wider to fit a wider foreshaft [48]–[51], and that penetration should be guaranteed by a greater size (and hence weight) rather than by velocity and tip angle [2]. Thus, wider stems, greater sizes, moderate asymmetries and greater tip angles are expected traits for this kind of spears.

Finally, hand-held tools or thrusting spears are free of the restrictions related to symmetry and tip angle, and its general attributes should enable durability, probably related to greater sizes, and strong attachment regions [3]. As points out the experimentation carried out by Odell and Cowan [51] with arrows versus hand-hurled spears, short and wide points with a plan view tip angle mean of 63° have a tendency to bounce off the target. In consequence, this sort of points does not work as flight weapon, being better as thrusting spears.

We have performed a composite PC analysis including all of these attributes previously studied as influencing the performance of particular point types. In general terms, the obtained results demonstrate that our composite sample display clear cases fitting well the above-described model. In particular, we detect a central role of tip angle, blade shape and general proportions of the point, followed by a secondary role of asymmetry and size. Of course, these relative contributions are applicable to our sample, and a different scenario can be obtained on different arrays of tools. However, an important result is that these traits tend to vary on an integrated fashion likely coincident with the physical expectations described above.

Even though many points fall into intermediate positions (see also ref. 9), probably because of variable reduction events, we detected three different morphologies which we labeled and submitted to a new modularity analysis. In simple words, and considering the functional predictions, our type 1 tools (largely coincident with “Bird V” tools) can be considered as points displaying the expected attributes of an arrow, whereas our type 2 points could be seen as tools displaying optimal thrown spear traits. Finally, we detected a small subgroup of four outlier points that differ from the type 1 and 2 patterns, mainly because of its greater size and asymmetry. They can be considered as thrusting or hand-held spears.

We argue, then, that since a composite sample like the one used here displays a coherent array of variables and grouping of elements coincident with mechanical expectations, classifications made on future specimens should take into account these specific attributes derived from experimental or ethnographic data.

If we take our grouping criteria as valid, the analyses of modularity performed independently on the three separate groups can be of help to understand the importance of reduction and its effect on the modular pattern of lithic points. This analysis (Figure 4) refine the modularity test performed on the total sample, and suggests that type 1 and 2 tools (e.g. arrows and thrown spears) should be viewed as a system formed by two near-decomposable units: the tip, and the rest of the projectile. Resharpening of the tools seems not to disrupt this pattern (table 2). Conversely, type 3 tools seem to work as a two-module system formed by the blade and the stem. Results concerning modularity on our type 3 group should be taken with caution, since they are based on a subsample of only four specimens. However, their outlying behavior is not only based on modularity, but also on the remainig morphometric attributes studied here.

This study is working from uncontrolled archaeological cases where the functional weapons delivery systems (arrows, thrown-spears, thrusting spears) are presumed but largely unknown. Future research must be done on ethnographical collections and experimental contexts in order to corroborate our predictions.

### Conclusions

A conclusion of our work is, thus, that to the classical attributes studied hereafter (size, shape, tip-angle, symmetry, blade/stem proportions, etc.), the modularity pattern exhibited by arrays of point samples can be of utility to infer lithic tools' past function. Modular design rules establish strict partitions of knowledge and effort at the outset of design. They are not just guidelines or recommendations: they must be rigorously obeyed in all phases of design and production. Operationally, this means that designers may not solve the problems of designing, constructing and maintaining the tip, by tweaking parameters affecting the stem. Because of the severe constraints it imposes, full-fledged modularity is never easy to achieve in practice. However, when implemented faithfully, modularity greatly reduces the costs of experimenting with new designs. An important connotation of the modularity concept applied to artifacts, from simple stone tools until modern informatics devices, is that it enhances the “evolvability” of complex systems by limiting the effects of technological innovation to sets of functionally related sub structures or “traits”. With modularity enforced, it is possible to change pieces of a system without redoing the whole [21]. This can be the explanation to the apparent high levels and ubiquity of resharpening practices, especially in contexts where the core material is scarce or economically expensive. In conclusion, stone tools can be seen as an example of designs becoming flexible and capable of evolving at the module level. This in turn creates new options for designers, and corresponding opportunities for innovation and competition in the realm of module design [21].

## Supporting Information

Table S1 **Further details concerning each point.** Classification under the Bird's system, qualitative and quantitative data regarding point's size, IBS, TA and asymmetry.
(XLSX)
